# Exploring pharmacist prescribing practices in general practices for atrial fibrillation in England: a qualitative study using the theoretical domains framework

**DOI:** 10.1007/s11096-025-02062-3

**Published:** 2025-12-10

**Authors:** Raman Sharma, Syed Shahzad Hasan, Barbara R. Conway, Muhammad Usman Ghori

**Affiliations:** Department of Pharmacy, School of Applied Sciences, University of Huddersfield, Huddersfield, UK

**Keywords:** Anticoagulants, Atrial Fibrillation, England, Pharmacists, Prescribing, Primary Health Care, Qualitative Research

## Abstract

**Introduction:**

Pharmacist roles in primary care are evolving, with increasing involvement in long-term condition management. An example is their crucial role in the management of atrial fibrillation (AF), particularly in prescribing and monitoring oral anticoagulation therapy. However, their experiences and challenges in this area remain underexplored, particularly within the context of general practice.

**Aim:**

This study aimed to explore the experiences, perceptions, and challenges of independent prescribing pharmacists when managing and prescribing for AF within general practice, using the Theoretical Domains Framework (TDF) to guide enquiry and analysis.

**Method:**

We conducted a qualitative study underpinned by the Theoretical Domains Framework (TDF), which informed both the interview guide and the analytic coding framework. Independent prescribing pharmacists working in general practice in England were purposively recruited via professional networks; eligible participants were patient-facing and had experience prescribing for atrial fibrillation. One-to-one, semi-structured interviews were conducted via Microsoft Teams® in August 2024, audio-recorded, transcribed verbatim, and returned to participants for checking. Recruitment proceeded until thematic saturation. Two researchers independently applied the framework method, resolved discrepancies by consensus, and mapped final themes to relevant TDF domains.

**Results:**

Twenty pharmacists took part in the study (9 men, 11 women; age 25–52 years), providing perspectives from a range of experience levels. Four overarching themes emerged: (1) confidence and experience in prescribing, (2) perceived role and responsibilities, (3) barriers to effective prescribing, and (4) strategies for effective prescribing. Pharmacists with extensive AF experience demonstrated higher confidence, whereas less experienced pharmacists relied on guidelines and colleagues. Perceived roles ranged from central to supportive within multidisciplinary teams, with some uncertainty about role boundaries. Key barriers included incomplete access to patient records, limited training, and workload pressures. Strategies to support prescribing included continuous professional development, decision support tools, and peer consultation.

**Conclusion:**

The study emphasises the challenges pharmacists encounter in managing AF, highlighting the need for clearer role definitions, improved access to patient data and ongoing peer support. Addressing the identified barriers through targeted interventions could enhance the effectiveness of pharmacist-led AF management in general practice. Future research should evaluate interventions designed to support pharmacists in this evolving role.

**Supplementary Information:**

The online version contains supplementary material available at 10.1007/s11096-025-02062-3.

## Impact statements

- This study identifies key barriers to effective pharmacist-led AF management, including unclear role boundaries and responsibilities, limited access to patient records, inadequate AF-specific training, and time pressures. These barriers map to TDF domains such as social/professional role and identity, knowledge, skills, social influences, and environmental context and resources.
- The findings highlight the need for enhanced support systems, including improved access to patient information and targeted continuing professional development, to empower pharmacists in their prescribing roles and to support optimal, multidisciplinary AF care.
- Addressing the identified barriers through targeted interventions, supported by policy changes and appropriate resource allocation, could significantly improve the integration of pharmacists into multidisciplinary teams, thereby enhancing the overall effectiveness of AF management in general practice settings.

## Introduction

Atrial fibrillation (AF) is the most common sustained cardiac arrhythmia and is associated with substantial stroke risk and increased mortality [1]. Globally, AF affects around 33 million people, with prevalence rising as populations age [2]. Optimal management is critical to reducing stroke risk and typically includes oral anticoagulation (OAC), rate or rhythm control and lifestyle modification [3].

OAC is the mainstay of stroke prevention and is recommended by the National Institute for Health and Care Excellence (NICE) [4]. Direct oral anticoagulants (DOACs) have demonstrated effectiveness in clinical practice and are now considered first-line therapy for most patients [4]. National initiatives have prioritised AF detection, OAC initiation and optimisation [5–7]; however, OAC remains underused in around one-third of eligible patients [8, 9] and is classified as high-risk medicine because of the potential for serious adverse drug reactions [10, 11].

Pharmacists play an increasingly important role within multidisciplinary AF care. Responsibilities have expanded beyond dispensing to include medicines optimisation, patient education and, in some settings, prescribing [12]. In the United Kingdom (UK), independent prescribing rights have enabled pharmacists, particularly in general practice, to assume greater responsibility for chronic disease management and influence treatment decisions and outcomes [13]. This shift aligns with national policy emphasising multidisciplinary approaches to long-term conditions [14]. The integration of pharmacists into general practice was intended to increase capacity and improve access to care, especially for patients with chronic diseases [12]. Primary Care Networks (PCNs) were established to build on earlier pilot programmes and aimed to place at least one pharmacist in every general practice by 2024 [15]. As of June 2024, 5,324 whole-time equivalent pharmacists were employed in PCNs across England, compared with 609 in June 2020 [16].

The breadth of the role of pharmacists in general practice has been described in several studies [17–19], highlighting activities such as medication reviews, medicines optimisation, long-term condition management and independent prescribing. The Structured Medication Review (SMR) service, introduced by NHS England in September 2020, was designed to utilise pharmacist capacity within PCN member practices to deliver SMRs for targeted populations, typically those with long-term conditions including cardiovascular disease [20].

Pharmacist-led AF care has been shown to improve OAC prescribing and bleed risk management in general practice [21]. A recent systematic review and meta-analysis reported that pharmacist-led AF management increased the appropriateness of OAC prescribing threefold compared with usual care [22]. Despite this emerging evidence base, little research has examined factors influencing independent prescribing pharmacist-led AF management in UK general practice. Existing work includes a qualitative study of pharmacist AF management in UK primary care with 11 participants, only four of whom worked in general practice and not all of whom were independent prescribers [23], and a qualitative study by Savickas et al. on pharmacist detection and screening of AF in general practice [24].

Pharmacy practice research has often focused on the design, implementation and evaluation of interventions to optimise outcomes and safety [25]. Given the growing evidence for pharmacist-led AF interventions [21, 22], these services clearly involve behavioural change on the part of pharmacists [26], yet provision remains limited and inconsistent [23]. Updated guidance from the UK Medical Research Council (MRC) on developing and implementing complex interventions highlights the importance of using theory, particularly behavioural theories, to understand barriers and enablers to implementation [27]. The Theoretical Domains Framework (TDF) synthesises key behavioural constructs and offers a comprehensive approach to understanding the behaviours and practices of healthcare professionals, particularly in relation to prescribing and management of chronic conditions such as AF [28].

### Aim

The aim of this study was to explore the experiences, perceptions and challenges of independent prescribing pharmacists in general practice when managing and prescribing for patients with AF, and to identify key contextual enablers and barriers to effective pharmacist-led AF management in primary care.

### Method

The study design, conduct and reporting adhere to the Consolidated Criteria for Reporting Qualitative Research (COREQ) [29], with the completed checklist provided in Supplementary File S1. A qualitative study design was employed to explore the experiences, perceptions, and challenges of independent prescribing pharmacists managing AF. This approach was selected to capture the depth and complexity of pharmacist prescribing practices and to understand enablers and barriers within their professional context. The TDF was used to guide both the development of the interview guide and the analysis, ensuring that findings were interpreted within a robust behavioural science framework.

### Participants and recruitment

Pharmacists working in a general practice setting and qualified as independent prescribers were eligible to participate in this study, with purposive sampling being employed. To be included in the study pharmacists had to be in a patient-facing role and have had some interaction with AF patients, including, but not limited to, initiation and reviewing OAC prescribing. Recruitment took place through the research team’s professional networks, with invitations sent via targeted emails and peer contacts. Prior to interviews, participants were provided with written information and consent forms about the study. Informed consent was provided by participant relating to their involvement in the study and the future dissemination of anonymised data. Prior to the commencement of the interview, each participant was required to provide signed consent that demonstrated their informed agreement, as detailed in the participant information sheet, Supplementary File S2.

### Reflexivity

The lead researcher (RS) is a clinical pharmacist and independent prescriber with prior experience in qualitative interviewing. To reduce bias, RS had no supervisory or line management relationship with participants. A reflexive log was maintained throughout data collection and analysis to document assumptions and reflections. Data coding was conducted by two researchers (RS and MUG) independently, with consensus reached in team meetings to enhance credibility.

### Data collection

Semi-structured interviews were conducted in August 2024 via Microsoft Teams®, at times convenient for participants. Sampling and the process of data generation were carried out until the data saturation and data adequacy were reached [30]. The initial analysis sample size of 10 interviews was determined at the outset with a stopping criterion of 3 adopted [31], defined as three consecutive interviews yielding no new codes. Coding and analytic memo-writing were carried out alongside data collection, commencing after the second interview, so that emerging concepts, negative cases and provisional code definitions could be documented and refined iteratively. A semi-structured interview schedule was developed, with the interview parameters being focused on prescribing for AF patients, with particular focus on OAC, and the 14 behavioural determinants of TDF, i.e. influences (knowledge, skills, professional role, beliefs about capabilities, optimism, beliefs about consequences, reinforcement, intentions, goals, decision processes, context of environment/resources, social influences, emotions, behavioural regulation) [32]. A piloted interview guide, mapped to the 14 domains of the TDF, ensured comprehensive coverage of behavioural influences [27, 33], and the full interview schedule is provided in Supplementary File S3. Two pilot interviews were undertaken and included in the analysis after refinement of interview prompts. Subsequent prompts were adjusted iteratively to probe emergent ideas while preserving TDF coverage. Senior researcher (MUG) analysed pilot interview recordings, offering input on interview methodology to enhance credibility and reliability [34].

### Data analysis

Following the interviews, all the transcripts were anonymised and examined for typographical errors and missing information. The following framework approach was used to analyse the transcripts: data familiarisation, establishing coding framework, indexing and charting, mapping, and interpretation. Behavioural influences, as per the TDF, were used as a coding framework for analysis, using Microsoft Excel®, with an overview of the coding tree available in Supplementary File S4. Two researchers (RS and MUG) coded each interview independently, and a consensus for the coding framework was reached at a research group meeting.

### Ethics approval

The study was granted ethics approval by the School of Applied Sciences Research Integrity and Ethics Committee, University of Huddersfield (SAS-SRIEC-06.08.24–1) on 6th August 2024.

## Results

### Demographic data

A total of 20 pharmacists participated in this study, representing a diverse range of backgrounds and experiences within general practice settings in England. All participating pharmacists work in a general practice setting with independent prescriber status. The participants included 9 males and 11 females, with ages ranging from 26 to 52 years. This range of demographics provided a broad perspective on the prescribing practices and challenges faced by pharmacists in managing AF. Participant demographic data is shown in Table 1. Interviews lasted 32–64 min (mean 48 min), were audio-recorded, and transcribed verbatim using the platform’s transcription function. Transcripts were returned to participants for checking; minor corrections were made where requested.

**Table 1:** Demographics of pharmacists included in the study

Participant	Age	Gender	Experience	
			Pharmacist (years)	Independent prescriber (years)
1	42	Male	16–20	6–10
2	38	Female	16–20	6–10
3	27	Female	1–5	1–5 y
4	34	Male	11–15	6–10
5	26	Male	1–5	1–5
6	30	Female	6–10	6–10
7	52	Female	20 +	6–10
8	28	Male	1–5	1–5
9	45	Female	16–20	6–10
10	47	Female	20 +	6–10
11	35	Female	6–10	6–10
12	31	Male	6–10	6–10
13	25	Male	1–5	1–5
14	29	Female	1–5	1–5
15	39	Female	11–15	6–10
16	41	Male	11–15	6–10
17	29	Male	6–10	6–10
18	37	Female	11–15	6–10
19	34	Male	6–10	6–10
20	28	Female	1–5	1–5

**Fig. 1:**
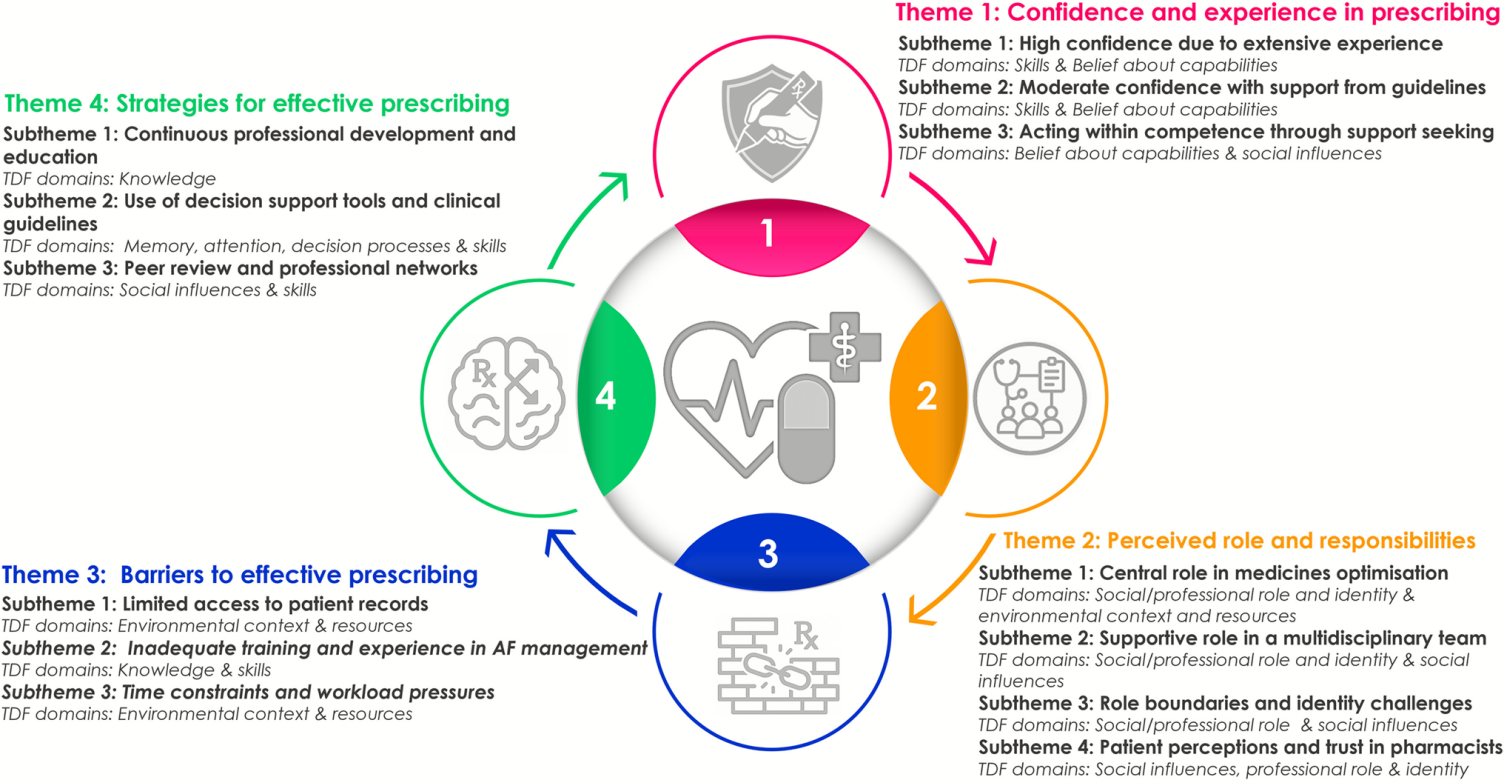
Summary of the four key themes and subthemes emerging from the qualitative analysis, mapped to relevant Theoretical Domains Framework (TDF) domains

Analysis of the interview data identified four main themes, confidence and experience in prescribing; perceived role and responsibilities; barriers to effective prescribing; and strategies for effective prescribing, each with corresponding subthemes. These encompassed variations in prescribing confidence, the diversity and clarity of pharmacist roles, practical challenges such as limited access to records and high workload, and the importance of ongoing professional development, decision support, and peer collaboration. The mapping of these themes and subthemes to relevant TDF domains is visually summarised in Fig. 1, demonstrating the multifaceted influences on pharmacist-led AF management.

In addition, Table 2 summarises the specific facilitators and barriers that emerged from the interviews, offering a clear comparison of the main factors that either supported or hindered effective prescribing practices among general practice pharmacists. The table presents these facilitators and barriers alongside their corresponding TDF domains, highlighting how factors such as access to training, role clarity, and peer support contributed positively, while challenges like limited guideline familiarity, time constraints, and lack of confidence posed significant obstacles to optimal pharmacist-led care.

**Table 2:** Key facilitators and barriers identified from pharmacist interviews mapped against the most relevant TDF domains

TDF domain	Facilitators	Barriers
Knowledge	Access to continuous professional development (CPD) and training.	Limited familiarity with up-to-date AF guidelines
Skills	Ongoing practice and experience with AF patients.	Limited AF-specific experience; acting within competence by seeking senior input for complex cases
Social/professional role	Clear definition and recognition of pharmacist prescribers, reinforced by GP endorsement, proactive patient-facing role communication, and brief co-consults for higher-risk decisions.	Unclear boundaries and limited role visibility, prompting patient preference for GP confirmation and inconsistent recognition of pharmacist prescribing
Beliefs about capabilities	Positive reinforcement from successful cases and colleague feedback.	Self-doubt in prescribing decisions, especially with anticoagulants
Environmental context/resources	Availability of decision support tools and peer support.	A busy clinical environment and lack of time to undertake comprehensive care in this patient group.

#### Theme 1 – Confidence and experience in prescribing

Confidence and experience in prescribing medications for AF emerged as a key theme in this study. The pharmacists' levels of confidence and experience were closely linked to several domains of the TDF, particularly knowledge, skills, beliefs about capabilities, and social influences.

##### Subtheme 1: High confidence due to extensive experience (mapped to TDF domains: skills, beliefs about capabilities)

Pharmacists with extensive experience in managing AF patients demonstrated a high level of confidence in prescribing, a belief in their abilities and well-developed skills which led to their confidence in managing AF patients and prescribing for them. This was evident in the responses of Pharmacist A, who, with 18 years of experience, described their confidence in prescribing:

"*I’ve had quite a lot of experience prescribing medications for patients, mainly focusing around AF and DOACs. I feel quite confident in being able to initiate the appropriate treatment for the patient.*" (P1).

In contrast, another participant described hesitancy when prescribing for AF, particularly around initiating DOACs in complex cases.

*“I feel less confident initiating DOACs in complex cases, where multimorbidity and competing risks make decisions challenging.” * (P7).

##### Subtheme 2: Moderate confidence with support from guidelines (mapped to TDF domains: knowledge, skills, beliefs about capabilities)

Pharmacists who reported moderate confidence often relied on their knowledge of clinical guidelines and the use of decision support tools to reinforce and support their prescribing decisions. This reliance on external resources helped bridge the gap between their knowledge and their belief in their capabilities.

*"I am fairly confident in prescribing for AF, but I always make sure to follow the NICE guidelines closely and use decision support tools to guide my decisions." * (P9).

##### Subtheme 3: Acting within competence through support seeking (mapped to TDF domains: beliefs about capabilities, social influences)

Pharmacists with less experience in AF management described feelings of uncertainty when making prescribing decisions. However, rather than being solely a matter of “low confidence,” these accounts often reflected appropriate professional judgement, recognising the limits of their current knowledge and seeking advice from more experienced colleagues. This illustrates how pharmacists managed risk by acting within their competence:

*"My experience in prescribing for AF is limited, and I often feel uncertain… I usually ask for advice from more experienced colleagues before making decisions." * (P5).

Another participant described navigating uncertainty by pairing guideline use with targeted case discussion:

*“I start with NICE and the PCN checklist to cover essentials, then confirm any uncertainties with the GP lead.” * (P8).

This behaviour highlights an important dimension of safe practice: although less experienced pharmacists may not independently initiate treatment, they are able to safeguard patient care by drawing on social support and available expertise until their competence develops.

#### Theme 2: Perceived role and responsibilities

All interviewees discussed at length the emergent second major theme. This theme focuses on how pharmacists perceive their roles and responsibilities in managing AF. Pharmacist perceptions of their roles varied, with some viewing themselves as central and proactive in the management of AF, while others saw their role as more supportive or adjunctive to other, more senior, healthcare professionals in their respective general practices.

##### Subtheme 1: Central role in medicines optimisation (mapped to TDF domains: social/professional role and identity, environmental context and resources)

Many pharmacists described a central role in medicines optimisation, aiming for guideline-concordant, target-driven anticoagulation delivered through longitudinal monitoring and review to maintain safety and effectiveness.

As one interviewee explained,

*"My role as a pharmacist generally involves managing the medication side of things… Ensuring that the patient is appropriately anticoagulated and that their treatment is being monitored regularly." * (P1).

Many described the importance of ongoing medication review to ensure appropriateness of therapy,

*“…part of the role is make sure to review and adjust treatment plans based on the individual patient in front of me, which is evolving over time, taking into account other health conditions and e.g. renal function as they age.” * (P15).

##### Subtheme 2: Supportive role in a multidisciplinary team (mapped to TDF domains: social/professional role and identity, social influences)

Many interviewees viewed their role as more supportive, working in collaboration with the wider practice teams of doctors and other healthcare professionals to manage AF. This perspective was often shaped by the collaborative nature of their work environment, where decisions are made jointly. Pharmacist C reflected this view:

*"I see my role as supporting the healthcare team, helping to ensure that patients are on the correct medication and understand their treatment, while working closely with the rest of the team to make changes to therapy."* (P3).

This coordination focus was echoed by mid-career prescribers, highlighting routine collaboration with GP leads within MDT pathways to maintain coherent, patient-centred anticoagulation plans.

*“I review anticoagulation, explain options to patients, then align any changes with the GP lead to keep plans consistent.”* (P11).

##### Subtheme 3: Role boundaries and identity challenges (mapped to TDF domains: social/professional role, identity and social influences)

Some pharmacists described challenges around role boundaries and professional identity, particularly when responsibilities for initiation versus monitoring were not explicitly defined within the practice. This sometimes led to uncertainty about responsibilities or hesitancy in making independent prescribing decisions. In addition to intra-team boundaries, several pharmacists perceived that some patients sought GP confirmation before accepting anticoagulation changes, reflecting variable recognition of pharmacist prescribing role and credibility: this occasionally delayed initiation despite agreement within the team.

As one interviewee explained,

*"There are times when it’s unclear whether I should be making the prescribing decision or if it’s better left to the GP… It can be challenging to work around these role boundaries."* (P20).

An experienced pharmacist linked hesitation to unclear responsibilities in high-risk scenarios, citing role ambiguity during DOAC initiation.

*“I sometimes struggle to initiate DOACs in complex multimorbidity; role boundaries aren’t always clear in critical cases.” * (P7)

##### Subtheme 4: Patient perceptions and trust in pharmacists (mapped to TDF domains: social influences, professional role and identity)

A few participants noted situations where patients were reluctant to accept their prescribing advice until it was confirmed by a GP. This highlighted not only the role boundaries within the healthcare team but also the influence of patient perceptions on pharmacist ability to enact their prescribing role.

Less frequently, some interviewees described situations to persuade patients to commence oral anticoagulation therapy,

*“I tried to convince them of the alternatives to warfarin that we have, explaining that it’s readily available, would not require the same regular monitoring at the hospital, it is as good, but sometimes they’re insisting on discussing this with the healthcare professional they regularly see at the warfarin clinic.”* (P17).

*“I recommended a patient start on a DOAC, gave them the options and discussed the differences between them. She seemed reluctant and said she would have a think about it. Later that week, the GP let me know she had made an appointment with him and asked about the DOACs and was happy for it to started by the doctor.”* (P19).

This suggests that while pharmacists may be clinically competent, patient trust and acceptance of their role can act as an additional barrier to effective prescribing.

#### Theme 3: Barriers to effective prescribing

Barriers to effective prescribing emerged as a core theme. Interviewees identified numerous challenges encountered that may hinder the effective management of AF patients.

##### Subtheme 1: Limited access to patient records (mapped to TDF domain: environmental context and resources)

A recurring barrier mentioned by pharmacists was the difficulty in accessing comprehensive patient records, which are crucial for making informed prescribing decisions. This lack of access often led to uncertainty and hesitation in prescribing, as highlighted by participant 4:

*"It’s really challenging when you don’t have full access to patient records as they patients have their blood tests and monitoring done elsewhere e.g. in the hospital and you do not always get to see the results… You’re making decisions with incomplete information, which isn’t ideal when prescribing anticoagulants and trying to review medications."* (P4).

Another participant, expressed this concern, noting how this barrier often delayed their decision-making process:

*"Sometimes, I have to wait for test results or information from other providers, which can delay the starting of treatment as often the diagnosis of AF is done at the hospital. This can be frustrating and can affect patient outcomes."* (P12).

##### Subtheme 2: Inadequate training and experience in AF management (mapped to TDF domains: knowledge, skills)

A number of interviewees expressed that their training and experience in AF management were inadequate.

*"I feel like my training didn’t fully prepare me for the complexities of AF management… There’s a lot I still need to learn, and that makes me hesitant to make clinical decisions."* (P14).

*"I often find myself requesting advice from the GP when it comes to making final decisions… I just don’t feel fully prepared to take on that responsibility yet."* (P3).

##### Subtheme 3: Time constraints and workload pressures (mapped to TDF domain: environmental context and resources)

Time constraints and high workload were also described as significant barriers that impacted pharmacists' ability to prescribe effectively.

*"There’s just not enough time to really dive into each patient’s case… You end up making quick decisions, which isn’t always the best approach, especially with something as critical as AF."* (P2).

Similarly, interviewees described how the workload affected their ability to follow up with patients:

*"I struggle to keep up with all the follow-ups, and that’s crucial for AF patients. The workload just doesn’t allow for it in and amongst other tasks that have to be done."* (P16).

*“Because of admin and same-day requests, I sometimes cannot perform proactive reviews, so anticoagulation reviews slip to next week.”* (P13).

#### Theme 4: Strategies for effective prescribing

Pharmacists employed various strategies to stay updated on the latest guidelines and make informed prescribing decisions.

##### Subtheme 1: Continuous professional development and education (mapped to TDF domain: knowledge)

A common approach among pharmacists was engaging in continuous professional development (CPD) activities, such as attending workshops, conferences and training sessions. These opportunities allowed them to stay informed about the latest guidelines and emerging treatments.

*"I make it a point to attend relevant CPD sessions and conferences whenever possible. It’s crucial to stay on top of the latest guidelines and treatments for AF."* (P1).

*"The CPD courses I attend, especially those focused on cardiovascular health, are incredibly useful. They help me stay confident in my prescribing choices."* (P4).

*“A recent anticoagulation webinar changed my approach, now I check renal trends first and document bleed risk more systematically.”* (P14).

##### Subtheme 2: Use of decision support tools and clinical guidelines (mapped to TDF domains: memory, attention, and decision processes, skills)

Many interviewees also relied heavily on decision support tools and clinical guidelines to inform their prescribing decisions. These resources provided quick access to evidence-based recommendations, helping pharmacists navigate complex cases with confidence.

*"I always use decision support tools and make sure to have the latest clinical guidelines on hand. They’re very useful when it comes to making safe prescribing decisions."* (P9).

*“The EHR prompts, and our anticoagulation checklist remind me about renal function, interactions, and bleeding risk so I don’t miss steps.”* (P18).

##### Subtheme 3: Peer review and professional networks (mapped to TDF domains: social influences and skills)

Several interviewees emphasised the importance of peer consultation and review and professional networks as crucial elements of their approach to staying informed and making well-informed decisions, alongside formal tools and education.

*"I often consult with my colleagues, especially when I’m unsure about a case. These discussions are invaluable for gaining new insights and ensuring I’m making the best decisions for my patients."* (P2).

One participant also highlighted the role of professional networks in staying informed:

*"Being part of a professional network allows me to stay in the loop with the latest practices in AF management. It’s a great way to learn from others’ experiences and discuss particular cases."* (P6).

## Discussion

### Statement of key findings

This study explored the experiences and perceptions of pharmacists regarding their role in prescribing medications for atrial fibrillation (AF) in England. Through qualitative analysis, four key themes emerged: *confidence and experience in prescribing; perceived role and responsibilities; barriers to effective prescribing; and clinical decision-making*. Pharmacists demonstrated varying levels of confidence, with those possessing greater experience often expressing more certainty in their prescribing capabilities. Perceptions of responsibility also varied; while some pharmacists viewed themselves as integral to the management of AF, others perceived their role as more supportive within the multidisciplinary team. Several significant barriers were identified, including limited access to patient records, inadequate training, and workload pressures. In response to these challenges, pharmacists adopted strategies such as continuing professional development (CPD), the use of decision support tools, and peer support to enhance their prescribing practice.

### Strengths and weaknesses

This qualitative study, underpinned by the TDF, used a structured framework to design the interview guide and analysis, systematically exploring perceptions, experiences and barriers to effective prescribing and situating findings within a robust theoretical model. Data saturation was achieved, and, given the exploratory/analytic aim (rather than formal theory generation), the sample provided sufficient information power for framework/thematic analysis. To the authors’ knowledge, this is the only qualitative study to date focusing exclusively on independent prescribing pharmacists in general practice managing AF.

Trustworthiness was enhanced through piloting the interview guide, concurrent data collection and analysis, and independent coding by two researchers, which supported credibility. An audit trail of coding decisions and analytic discussions promoted dependability. Reflexivity and peer debriefing contributed to confirmability, while rich contextual descriptions support transferability to similar general practice settings. Limitations include the focus on a single professional group within one national healthcare system, with participants drawn from PCNs in Yorkshire and Lancashire, England, which may limit wider applicability. Variations in local AF pathways may have shaped experiences, and the use of self-reported data introduces the potential for social desirability bias. However, these limitations are balanced by the breadth of participant demographics and the strong theoretical underpinning of the analysis.

### Interpretation

The expanding role of pharmacists in long-term condition management continues to create challenges in defining roles and responsibilities in clinical practice [12, 35, 36]. Participants reported that this lack of clarity is not limited to AF but also affects other clinical areas. Savickas et al. found that the way pharmacists see their role in AF management is shaped by the practice setting, relationships with other healthcare professionals and inconsistent role definitions [24]. This ambiguity can lead to hesitation in independent prescribing and reflects wider problems in defining professional roles within multidisciplinary teams [37]. Patients’ views of the role of the pharmacist prescriber also influenced whether they accepted anticoagulation recommendations, which links to the TDF domains of social influences and professional role and identity.

Barriers to effective prescribing, especially limited access to patient records in areas with different AF care models, were major concerns for several pharmacists and are consistent with evidence that fragmented care pathways can harm patient outcomes [38–40]. A heavy workload was also seen as a major barrier to good care, with time pressures widely recognised as having a negative impact on care quality and clinical decision-making in many healthcare professions [41].

Strategies used by pharmacists to stay up to date and make informed decisions, such as using decision support tools and following local and national clinical guidelines, are widely recognised as ways to support evidence-based practice and its implementation [42]. Previous qualitative studies also report that pharmacists use these approaches to carry out CPD and develop their skills in AF management [24].

### Further research

The findings from this study suggest several avenues for further research. Future work should examine the transferability of these findings across diverse care contexts where AF pathways differ (e.g., varying PCN arrangements, shared care with cardiology and anticoagulation clinics). Further work should investigate community and hospital pharmacy settings to clarify their current roles in AF management and interfaces with general practice. Incorporating the perspectives of patients and other healthcare professionals could deepen understanding of multidisciplinary AF care, whilst co-developing and evaluating targeted, theory-informed interventions to address the barriers identified in this study.

## Conclusion

This qualitative study identified key behavioural determinants influencing pharmacist prescribing practices for atrial fibrillation (AF), categorised into four main themes: confidence and experience in prescribing; perceived role and responsibilities; barriers to effective prescribing; and clinical decision-making. The findings highlight that prescribing confidence is shaped by pharmacist clinical experience and access to ongoing professional development. Significant barriers, including limited access to patient records, time constraints and insufficient training were found to hinder effective prescribing and consistent care delivery. Addressing these challenges requires clearer role definitions, improved access to clinical information and enhanced training and support structures within general practice. Further research is needed to explore targeted interventions to overcome these barriers.

## Supplementary Information

Below is the link to the electronic supplementary material.

Supplementary file1 (DOCX 25 kb)

Supplementary file2 (DOCX 16 kb)

Supplementary file3 (DOCX 21 kb)

Supplementary file4 (DOCX 19 kb)

## Data Availability

The datasets generated and analysed during the current study are not publicly available due to participant confidentiality but are available from the corresponding author on reasonable request.
