# Supplementary material for: Exploring pharmacist prescribing practices in general practices for atrial fibrillation in England: a qualitative study using the theoretical domains framework
Source: Int J Clin Pharm. 2025 Dec 10;48(3):740–50. doi: 10.1007/s11096-025-02062-3 (PMC13176180; doi:10.1007/s11096-025-02062-3)
Supplement: Supplementary file 1 — Supplementary file1 (DOCX 25 kb) [file 11096_2025_2062_MOESM1_ESM.docx]

**Supplementary Material 1 – COREQ Checklist**

Consolidated criteria for reporting qualitative studies (COREQ): 32-item checklist

| **No. Item** | **Guide questions/description** | **Notes** |
| --- | --- | --- |
| **Domain 1: Research team and reﬂexivity** | | |
| ***Personal Characteristics*** | | |
| 1. Interviewer/facilitator | Which author/s conducted the interview or focus group? | RS conducted all interviews. |
| 2. Credentials | What were the researcher’s credentials? E.g. PhD, MD | MPharm, Msc |
| 3. Occupation | What was their occupation at the time of the study? | Clinical pharmacist and academic researcher at the time of the study. |
| 4. Gender | Was the researcher male or female? | Male |
| 5. Experience and training | What experience or training did the researcher have? | Experienced clinical pharmacist, trained in qualitative research interviewing, piloted the interview schedule. |
| *Relationship with participants* |  |  |
| 6. Relationship established | Was a relationship established prior to study commencement? | No prior relationships with participants before recruitment. |
| 7. Participant knowledge of the interviewer | What did the participants know about the researcher? e.g. personal goals, reasons for doing the research | Participants were informed that RS was a pharmacist researcher exploring AF prescribing in general practice. |
| 8. Interviewer characteristics | What characteristics were reported about the inter viewer/facilitator? e.g. Bias, assumptions, reasons and interests in the research topic | Reflexive statement included; RS acknowledged shared professional background as a pharmacist, with potential assumptions mitigated through independent coding and peer debriefing. |

| **Domain 2: study design** | | |
| --- | --- | --- |
| ***Theoretical framework*** | | |
| 9. Methodological orientation and Theory | What methodological orientation was stated to underpin the study? e.g. grounded theory, discourse analysis, ethnography, phenomenology, content analysis | Qualitative study using framework analysis, underpinned by the Theoretical Domains Framework (TDF). |
| ***Participant selection*** | | |
| 10. Sampling | How were participants selected? e.g. purposive, convenience, consecutive, snowball | Purposive sampling. |
| 11. Method of approach | How were participants approached? e.g. face-to-face, telephone, mail, email | Invitations sent via professional networks and targeted emails. |
| 12. Sample size | How many participants were in the study? | 20 participants. |
| 13. Non-participation | How many people refused to participate or dropped out? Reasons? | Not systematically recorded; of those expressing interest, 20 were interviewed. |
| ***Setting*** | | |
| 14. Setting of data collection | Where was the data collected? e.g. home, clinic, workplace | Remote interviews conducted via Microsoft Teams®. |
| 15. Presence of non-participants | Was anyone else present besides the participants and researchers? | No one else present during interviews. |
| 16. Description of sample | What are the important characteristics of the sample? e.g. demographic data, date | Demographic data (age, gender, years of experience, practice setting) reported in Table 1. |
| ***Data collection*** | | |
| 17. Interview guide | Were questions, prompts, guides provided by the authors? Was it pilot tested? | Semi-structured interview schedule developed and piloted; provided as Supplementary File 1. |
| 18. Repeat interviews | Were repeat interviews carried out? If yes, how many? | None conducted. |
| 19. Audio/visual recording | Did the research use audio or visual recording to collect the data? | Audio-recorded via Microsoft Teams® with automatic transcription. |
| 20. Field notes | Were ﬁeld notes made during and/or after the inter view or focus group? | A reflexive log and notes were maintained during and after interviews. |
| 21. Duration | What was the duration of the interviews or focus group? | 32–64 minutes (mean 48 minutes). |
| 22. Data saturation | Was data saturation discussed? | Yes, using stopping criterion of 3 consecutive interviews with no new codes. |
| 23. Transcripts returned | Were transcripts returned to participants for comment and/or correction? | Yes, participants were offered their transcripts; minor corrections were made where requested. |
| **Domain 3: analysis and ﬁndings** | | |
| ***Data analysis*** | | |
| 24. Number of data coders | How many data coders coded the data? | Two (RS and MUG). |
| 25. Description of the coding tree | Did authors provide a description of the coding tree? | Yes, provided as Supplementary File 3. |
| 26. Derivation of themes | Were themes identiﬁed in advance or derived from the data? | Themes were derived inductively from data, mapped to TDF domains. |
| 27. Software | What software, if applicable, was used to manage the data? | Microsoft Excel® |
| 28. Participant checking | Did participants provide feedback on the ﬁndings? | No formal member-checking of findings; transcript validation only. |
| ***Reporting*** | | |
| 29. Quotations presented | Were participant quotations presented to illustrate the themes/ﬁndings? Was each quotation identiﬁed? e.g. participant number | Yes, participant quotes included and labelled by ID. |
| 30. Data and ﬁndings consistent | Was there consistency between the data presented and the ﬁndings? | Yes, findings supported by participant data. |
| 31. Clarity of major themes | Were major themes clearly presented in the ﬁndings? | Yes, four major themes clearly reported. |
| 32. Clarity of minor themes | Is there a description of diverse cases or discussion of minor themes? | Yes, subthemes described, with diverse perspectives included. |
