# Supplementary material for: Exploring pharmacist prescribing practices in general practices for atrial fibrillation in England: a qualitative study using the theoretical domains framework
Source: Int J Clin Pharm. 2025 Dec 10;48(3):740–50. doi: 10.1007/s11096-025-02062-3 (PMC13176180; doi:10.1007/s11096-025-02062-3)
Supplement: Supplementary file 2 — Supplementary file2 (DOCX 16 kb) [file 11096_2025_2062_MOESM2_ESM.docx]

**Supplementary Material 2 – Participant Information Sheet**

**Participant Information Sheet**

**Title of Study:** Exploring Pharmacist Prescribing in Atrial Fibrillation (AF) Management

**Principal Investigator:** Mr Raman Sharma

**Introduction:**

Thank you for considering participation in our research study. Before you decide to take part, we want to ensure that you have all the information you need to make an informed decision. Please take some time to read this Participant Information Sheet carefully.

**Purpose of the Study:**

The purpose of this study is to gain a deeper understanding of the factors that influence pharmacist prescribing practices in the management of atrial fibrillation (AF) within general practices in England. We aim to explore pharmacists' knowledge, beliefs, skills, and experiences, as well as the social, environmental, and emotional factors that may impact their prescribing decisions.

**Study Procedures:**

If you agree to participate, you will be invited to take part in an interview with one of our research team members. During the interview, you will be asked open-ended questions about your experiences and perspectives related to pharmacist prescribing in AF management. The interview is expected to last approximately 60 minutes, and it will be conducted at a mutually convenient time and location.

**Confidentiality:**

Your privacy and the confidentiality of your responses are of utmost importance to us. All data collected will be treated with strict confidentiality. Your name and any personally identifiable information will not be linked to your responses. Data will be securely stored, and access will be restricted to authorized research personnel only. Your participation and responses will be anonymized and aggregated to protect your identity.

**Voluntary Participation:**

Participation in this study is entirely voluntary. You are under no obligation to take part, and your decision will not affect your professional status or relationship with your workplace or colleagues in any way.

**Withdrawal:**

You have the right to withdraw from the study at any time without providing a reason. Your decision to participate or withdraw will not result in any negative consequences.

**Benefits and Risks:**

There are no direct benefits or risks associated with participating in this study. Your input will contribute to our understanding of pharmacist prescribing practices in AF management, potentially leading to improvements in healthcare practices.

**Contact Information:**

If you have any questions or concerns about the study, you are encouraged to contact the Principal Investigator, Dr MU Ghori, at the Department of Pharmacy, School of Applied Sciences, University of Huddersfield, via email [m.ghori@hud.ac.uk](mailto:m.ghori@hud.ac.uk) or 01484 256950.

**Consent:**

If you decide to participate in the study, you will be asked to provide written consent before the interview. This consent form will clearly outline the terms of your participation.

**Thank you:**

We appreciate your consideration of participation in our research study. Your insights are valuable, and they will contribute to advancements in the field of pharmacist prescribing and atrial fibrillation management.

By participating, you acknowledge that you have read and understood the information provided in this Participant Information Sheet.
