# Supplementary material for: Exploring pharmacist prescribing practices in general practices for atrial fibrillation in England: a qualitative study using the theoretical domains framework
Source: Int J Clin Pharm. 2025 Dec 10;48(3):740–50. doi: 10.1007/s11096-025-02062-3 (PMC13176180; doi:10.1007/s11096-025-02062-3)
Supplement: Supplementary file 3 — Supplementary file3 (DOCX 21 kb) [file 11096_2025_2062_MOESM3_ESM.docx]

**Supplementary Material 3 – Interview Schedule/Guide**

Interview Schedule: Exploring Pharmacist Prescribing in Atrial Fibrillation (AF) Management

***Introduction:*** *Thank you for participating in this interview. The purpose of this interview is to gain a deeper understanding of the factors that influence pharmacist prescribing practices in the management of atrial fibrillation within general practices in England. Please share your thoughts and experiences openly.*

**Demographic Information:**

1. Age:
2. Gender:
3. Years of experience as a pharmacist:
4. Practice setting:

**Knowledge and Skills:**

1. Can you describe your knowledge and understanding of atrial fibrillation (AF) management guidelines in England?
2. Please share your experience and confidence in prescribing medications for patients with AF.

**Social/Professional Role and Identity:**

1. How do you perceive your role in managing atrial fibrillation in patients as a pharmacist?

**Beliefs about Capabilities:**

1. How confident are you in your ability to prescribe medications and provide recommendations for patients with AF? Can you provide an example of such a recommendation or prescription you have made?

**Optimism:**

1. How optimistic are you about the effectiveness of pharmacist-prescribed treatments for AF? Can you share any success stories or challenges related to this?

**Beliefs about Consequences:**

1. What do you believe are the positive outcomes of pharmacist prescribing in AF management?
2. What do you perceive as potential negative consequences or challenges associated with pharmacist prescribing in AF management?

**Reinforcement:**

1. Are there any external factors or incentives that influence your decision to prescribe medications for AF?

**Intentions:**

1. Can you describe your intentions regarding the frequency of prescribing medications for AF in the upcoming year?

**Goals:**

1. Do you have any specific goals or objectives related to pharmacist prescribing in AF management?

**Memory, Attention, and Decision Processes:**

1. How do you typically stay updated on the latest developments and guidelines in AF management that might influence your prescribing decisions?

**Environmental Context and Resources:**

1. What environmental factors or resource availability do you think impact your ability to prescribe effectively for AF?

**Social Influences:**

1. Are there any colleagues or peers who influence your decisions regarding pharmacist prescribing for AF? Can you provide examples?

**Emotion:**

1. How do you feel about pharmacist prescribing for AF? Are there any emotions or concerns that influence your decisions in this regard?

**Behavioural Regulation:**

1. Can you describe any strategies or processes you use to regulate your prescribing behaviour for patients with AF?

***Additional Comments:***

1. Do you have any additional comments or insights you would like to share regarding pharmacist prescribing in AF management?

***Closing:***

Thank you for sharing your valuable insights and experiences. Your input is instrumental in advancing our understanding of pharmacist prescribing practices in atrial fibrillation management.
