# Supplementary material for: Exploring pharmacist prescribing practices in general practices for atrial fibrillation in England: a qualitative study using the theoretical domains framework
Source: Int J Clin Pharm. 2025 Dec 10;48(3):740–50. doi: 10.1007/s11096-025-02062-3 (PMC13176180; doi:10.1007/s11096-025-02062-3)
Supplement: Supplementary file 4 — Supplementary file4 (DOCX 19 kb) [file 11096_2025_2062_MOESM4_ESM.docx]

**Supplementary Material 4 – Coding Tree**

| Theme | Subthemes / Codes | Illustrative TDF Domains |
| --- | --- | --- |
| Understanding and Knowledge (UK) | Knowledge of AF guidelines (KAFG), Strategies for staying updated (SSU) | Knowledge, Skills |
| Prescribing Confidence and Experience (PCE) | Confidence in prescribing (CP), Experience with AF patients (EAF), Challenges in prescribing (CHP) | Beliefs about capabilities, Skills |
| Perceived Role and Responsibilities (PRR) | Role perception (RP) | Professional role & identity |
| Emotional and Social Influences (ESI) | Emotional response (ER), Social influences (SI) | Emotion, Social influences |
| External Factors and Resources (EFR) | Environmental factors (EF), Incentives/resources (IR) | Environmental context & resources |
| Strategies and Processes (SP) | Strategies for prescribing (SPP) | Behavioural regulation, Decision processes |

The coding tree was developed iteratively. Initial codes were mapped to six overarching themes, which were then aligned with relevant domains of the Theoretical Domains Framework (TDF). Illustrative participant quotes for each theme are provided in the Results section of the manuscript.
